# Patients, antibiotics and *Clostridioides difficile*: when your best friend becomes your worst enemy

**DOI:** 10.1093/jacamr/dlaf163

**Published:** 2025-09-19

**Authors:** Johan Karp, Gunnar Jacobsson, Fredrika Sundberg

**Affiliations:** Department of Infectious Diseases, Skaraborg Hospital, Lövängsvägen, Skövde 451 42, Sweden; Centre for Antibiotic Resistance Research (CARe), Department of Infectious Diseases, Institute of Biomedicine, Sahlgrenska Academy, University of Gothenburg, Box 440, Gothenburg 405 30, Sweden; Skaraborgsinstitutet, Regionens hus, Skövde 541 80, Sweden; Department of Infectious Diseases, Skaraborg Hospital, Lövängsvägen, Skövde 451 42, Sweden; Centre for Antibiotic Resistance Research (CARe), Department of Infectious Diseases, Institute of Biomedicine, Sahlgrenska Academy, University of Gothenburg, Box 440, Gothenburg 405 30, Sweden; Centre for Research and Development, Skaraborg Hospital Skövde, Skövde 541 85, Sweden; The School of Health Sciences, University of Skövde, Högskolevägen, Box 408, Skövde 541 28, Sweden

## Abstract

**Background and objectives:**

Patients with recurrent *Clostridioides difficile* infection (rCDI) suffer long after infection. However, knowledge is incomplete regarding their perceptions of in-hospital care, antibiotic treatment and the most common risk factors for rCDI. Valid information processes are crucial for enabling such patients to avoid the suboptimal use of healthcare services and gain health literacy, good health and quality of life. This study aims to explore rCDI patients’ perceptions of the causes of rCDI, information processes and strategies for the future.

**Methods:**

Ten interviews, conducted with rCDI patients based on open-ended questions, were analysed using qualitative content analysis.

**Results:**

All the respondents identified antibiotic treatment as a trigger of rCDI, but they were unaware of other important factors. They also reported receiving inadequate information, often entirely absent or delivered by healthcare professionals under great pressure, together with other barriers that prevented successful information transmission. In addition to a lack of proper information, they described self-created barriers, some of which were severely counterproductive or even harmful, such as avoiding healthcare when they were ill or not collecting prescribed antibiotics due to fear.

**Conclusions:**

The respondents were often left to seek information and develop explanatory models, strategies and behaviours for the future by themselves. We recommend more standardized and personalized patient information to support effective preventive behaviours and to foster a better understanding of one of the most important tools of modern medicine—antibiotics.

## Introduction

*Clostridioides difficile* (*C. difficile*) infection (CDI) is an extremely common infection that causes suffering for patients and increases healthcare costs for society.^1,2^ Bacterial colonization of the gut often occurs during hospital stays^3^ since bacteria sporulate^4^ and easily survive on surfaces, and colonization due to the disturbance of gut microbiota by antibiotics can trigger the onset of disease. The spectrum, elimination and mechanism of action of an antibiotic can increase the risk of the drug causing CDI.^5,6^ Even long after infection, patients report that the debilitating conditions associated with CDI and recurrent CDI (rCDI) affect their physical, psychological, social and professional functioning.^7^ Moreover, regarding *C. difficile* colonization, 63% of the respondents in a Canadian study hesitated to seek hospital care and 17% hesitated to accept antibiotic treatment.^8^ Awareness of the connection between antibiotic treatment and the onset of CDI was high among CDI patients in an American study that addressed that topic.^9^

Healthcare professionals often underestimate the importance of providing sufficient information for patients,^10^ although well-informed patients are crucial for ensuring patient-centred care and shared decision-making.^11^ Patients need to acquire health literacy through information and communication with healthcare professionals before they can take an active part in their care and not see it as a burden.^12^ Scientifically tested communication strategies can be organized into four categories: cognitive aid strategies, persuasive strategies, relationship-oriented strategies and objectivity-oriented strategies.^13^ However, misinformed patients can experience poor health and quality of life^14^ and are associated with the suboptimal use of healthcare services^15^ and higher healthcare costs.^16^

The aim of this study was to explore rCDI patients’ perceptions of the causes of the disease, focusing on information processes and strategies for the future.

## Patients and methods

### Ethics

The Swedish Ethical Review Authority authorized this study (ref. 2023-01406-01). The participants were given written and oral information about the study on two separate occasions before signing written consent forms. They were also informed about their right to withdraw from participation at any time without giving an explanation.

### Participants

Eligible participants were selected via convenience sampling from a list of patients who had positive nucleic acid amplification test (NAAT) results confirming CDI. The list was provided by a local laboratory in southwestern Sweden between 1 January 2021 and 30 June 2023. Exclusion criteria were non-recurrent disease (with recurrent disease defined as a second positive test within 2–8 weeks after treatment completion combined with symptoms), age < 18 years, dementia and the need for a Swedish translator. The timeframe was selected to ensure that the patients would no longer be actively suffering from the disease but would still have vivid memories of their experiences. Letters, information about the study and consent forms were sent to 10 patients. The first author contacted them by phone 1–2 weeks later to verify their interest to participate, book appointments and answer questions regarding the study. Eight of the contacted patients agreed to participate. Therefore, three additional patients were contacted via the same procedure, two of whom agreed to participate. A total of 10 interviews were conducted with patients whose ages ranged from 52 to 79 years (six female and four male).

### Data collection

The data for this study were collected via individual semi-structured interviews, following a predetermined interview guide with open-ended questions relating to the aim. The interview guide was tested in two pilot interviews and analysed by the first and last authors. Since it did not require modification, these interviews were included in the analysis. The interviews were conducted at the respondents’ homes (seven interviews) or at the hospital (three interviews) according to the respondents’ preferences, and follow-up questions were asked based on their initial answers. The mean interview duration was 1 h. All interviews were conducted in person, recorded digitally and later transcribed by the first author, which generated 97 pages of text.

### Analysis

The data were analysed using qualitative content analysis and an inductive approach according to Elo and Kyngäs.^17^ The first and last authors read the interview transcripts several times to familiarize themselves with the material. The analysis was conducted by repeatedly identifying codes relating to the aim to ensure that no information was omitted. Then, the codes were grouped and categorized according to common content and abstracted into three generic categories, with coherent subcategories under each category. The first author conducted all these analytical steps, as described by Elo and Kyngäs.^17^ Finally, all the authors read the material again to ensure that the abstraction process respected the respondents’ meanings and fostered trust.

## Results

The analysis of the transcribed interviews resulted in three categories with subcategories, as shown in Table 1.

**Table 1.** Categories and subcategories from the analysis

Categories	Subcategories
Perceptions of the factors that cause rCDI	The role of antibiotics
	Risk factors for CDI
Receiving information about the disease	Ways of receiving information
	Perceptions of information given
	Barriers that prevent successful information delivery
	Suggestions for improving the provision of information by healthcare staff
Thoughts about the future	Concerns about future needs for healthcare and antibiotics
	Strategies for dealing with future needs for healthcare and antibiotics

### Perceptions of the factors that cause rCDI

In the interviews, the respondents reported their perceptions of the factors that cause rCDI. Many identified antibiotic treatment as a trigger, and some mentioned other risk factors. The respondents also knew that the disease was caused by a bacterium, but few understood how this bacterium had entered their guts.

#### The role of antibiotics

All respondents stated that antibiotic treatment caused their rCDI. Some of them described it as the sole cause of their disease, while others mentioned additional contributing factors. They believed that antibiotics destroyed the natural flora of the gut, which were often referred to simply as ‘good bacteria’. Some respondents also mentioned the opportunistic behaviour of *C. difficile* bacteria: ‘Antibiotic treatment deals with the bacteria, but when it kills many good bacteria, this mean little rascal (*C. difficile*) is given too much space’ (I1, female).

The respondents had diverse ideas about different antibiotics, often claiming that all antibiotics pose the same risk of rCDI and that the choice of antibiotic is unimportant. Another perception was that broad-spectrum antibiotics pose a greater risk of rCDI because they kill more ‘good bacteria’ than narrow-spectrum antibiotics. Some respondents also discussed the administration of antibiotics. Intravenous administration was deemed to pose both a higher and a lower risk of rCDI, as well as to pose the same risk as oral administration. One belief was that because intravenous administration bypasses the stomach, it has less impact on the gut. Another notion was that intravenous antibiotics are ‘stronger’ and therefore more harmful to the gut. One respondent believed that an antibiotic administered three times per day posed more risk than a single dose. There was also a perception that exposure to antibiotics inevitably weakens the immune system: ‘I have read that you can get this infection if you take too many antibiotics. It (the antibiotic) makes the body weaker, and these bacteria (*C. difficile*) then become so strong that they take over the good bacteria’ (I10, female).

#### Risk factors for rCDI

Four of the respondents concluded that a bacterium (*C. difficile*) was responsible for their rCDI, and most considered this bacterium to be a normal part of the gut flora in every individual: ‘Everyone has them! Everybody has *Clostridium difficile* in their stomach, or in their gut flora. But, as I said, all bacteria must be in harmony!’ (I9, male).

Another perception was that patients who developed rCDI had been infected with the bacterium via food, as with other forms of gastroenteritis. Other risk factors mentioned were older age, lack of exercise, lack of a varied diet and taking other drugs.

### Receiving information about the disease

The perception of most respondents was that information was vital for gaining an understanding of the condition they were suffering from. However, they described the information being given in different ways, and their opinions about the efficiency of information delivery varied greatly. Some respondents relied on healthcare personnel to deliver correct and sufficient information, while others sought information themselves.

#### Ways of receiving information

Some respondents received information in both oral and written form, some received information by telephone and some received no information at all. Some of the participants reported seeking information themselves, or with the help of relatives, and reading information about their condition online. The timing of the information given also varied widely, sometimes being given during hospital ward rounds, sometimes upon discharge from hospitals and sometimes over the phone when the respondents called healthcare professionals to ask questions.

#### Perceptions of information given

One respondent reported that the information given was inadequate or missing. Giving information was perceived as a low priority for healthcare professionals with limited resources. ‘It’s quite chaotic because there are no free beds. You get placed on the wrong ward, and healthcare professionals are very unsure about how to handle this condition. No, I received no information about what to do or why this had happened to me’ (I1, female).

Most of the respondents were dissatisfied with this situation, but a few mentioned that the information was not needed, and a few others were satisfied with the information given, describing it as correct, easy to understand and provided to an adequate extent. The respondents also claimed that they were given opportunities to ask questions if they wanted to.

#### Barriers to successful information

Sometimes, the respondents suffering from rCDI were too ill at the time to understand and remember the information given. They also explained that grounded trust in the healthcare system prevented them from questioning the care they received or asking questions about their disease. Furthermore, some respondents claimed that healthcare professionals so weighed down by a lack of resources have little time to give information. Some felt that healthcare professionals’ preconceived impressions (prejudices) hindered good communication with their patients. This included preconceived interpretations of the respondents’ symptoms, which led to broken trust between the respondents and representatives of the healthcare system, which made the information delivery process unsatisfactory: ‘I went to the hospital, to the emergency room, and tried to get help, but I was given a label—irritable bowel syndrome—placed here (pointing to the forehead)’ (I8, female).

#### Suggestions for improving information delivery processes

A common suggestion was that information should be given in both written and oral forms. Some respondents wanted their close relatives to be present to support them and help with memorizing the information, whereas others wanted them absent so that they would not worry. The respondents desired information about the causes of rCDI, lifestyle changes and what to eat to improve their conditions, preferably delivered in person or, if necessary, over the phone.

### Thoughts about the future

The respondents’ thoughts about the future revealed two opposing perspectives. Some described worries and strategies for dealing with their condition, which sometimes controlled their daily lives. Others claimed that healthcare personnel knew what was best for them and that they felt safe in their hands.

#### Concerns about future needs for healthcare and antibiotics

None of the respondents expressed worries about future hospital inpatient care. However, many expressed concern about further needs for antibiotics to varying degrees. One respondent claimed that she experienced daily worries and severe anxiety when thinking about the need for future antibiotic treatment: ‘I am really scared about being given this treatment again. I get, you know, this feeling of panic. My doctor prescribed another penicillin-based drug, but I didn’t collect it. I didn’t dare to take it’ (I8, female).

Some respondents worried about specific antibiotics causing rCDI episodes. They were more worried about antibiotics prescribed by primary care centres than hospitals, indicating greater trust in the qualifications of hospital-based doctors, but most respondents said that their trust was equal for both institutions.

#### Future needs for healthcare and antibiotics

One respondent stated that he would be reluctant to use toilets in hospitals. He claimed that he avoided public toilets and did not use the family bathroom at home, even though they had two; instead, he used a bucket in the garage. Concerning future needs for antibiotic treatment, the most drastic strategy was to avoid healthcare completely when ill to avoid the risk of antibiotic exposure. Other more moderate strategies mentioned were to inform the doctor about their rCDI history and request a ‘gentler’ antibiotic, to never accept an antibiotic treatment without consulting the Department of Infectious Diseases and to prepare their guts by taking probiotics: ‘I tell the doctor quite frankly, “We need to check this with the infectious diseases department first. Maybe you should choose another antibiotic in this case? You can do a quick culture in the blink of an eye and then choose a narrower-spectrum antibiotic that hits bulls’ eye!”’ (I9, male).

## Discussion

The most important findings of this study were insights concerning the information processes between healthcare professionals and rCDI patients. Regarding information processes in general, the respondents’ descriptions were diverse. Although the respondents in this study were from the same hospital in southwestern Sweden, it was clear that the information they received was neither standardized nor personalized. The respondents claimed that information was squeezed in if time was available, but that it rarely was. If healthcare professionals fail to give sufficient information to patients, patients will inevitably develop strategies for themselves, as shown by a telephone survey of rCDI patients conducted in 2017.^18^ As described previously, some of these strategies are effective, some are expensive and some are severely counterproductive.

Standardized written information based on guidelines—an objectivity-oriented information strategy^13^—would provide a common knowledge base and a minimum level of correct information for all patients suffering from rCDI (i.e. what to inform patients about). It could also serve as a foundation for more personalized information sharing between patients and healthcare professionals that would supplement, elaborate on and individualize the written information. This oral information would be categorized as a relationship-oriented information delivery strategy, focused on *how* to inform patients, which is essential for achieving patient-centred care (i.e. patients taking an active part in planning their care and developing strategies for the future in collaboration with healthcare professionals).^12^

We believe that objectivity-oriented and relationship-oriented strategies are complementary and equally essential when providing information to rCDI patients. We recommend that the information given to these patients should be standardized and delivered in both written and oral forms through what we call a *bilateral information delivery process.* Bilateral means that both patients and healthcare professionals must be actively involved in facilitating person-centred care and bilaterally focused on *how* and *what* information should be delivered. This would allow healthcare professionals to foster preventive behaviours in patients and to ensure that these behaviours are effective (i.e. not unsuccessful or harmful, as some examples from our interviews showed). It is unlikely that patients would implement all the advice given, as shown by an earlier study,^19^ but this routine would provide good basic conditions for helping patients develop practices for ensuring future lives without rCDI.

We plan to develop patient information tailored to the Swedish context, based on the findings of this study and current guidelines. We also plan to test the bilateral information delivery process with this patient information through an implementation study.

In this study, we aimed to explore and describe rCDI patients’ perceptions of the causes of the disease. The respondents’ perceptions of the causes of rCDI were diverse, covered various supposed risk factors and prompted them to develop diverse preventive behaviours. Some of these behaviours aligned with the literature and expert opinions concerning the pathophysiology of rCDI, while others lacked support, and some could be harmful, or even lethal, for the patients.

The strategy one respondent mentioned—avoiding healthcare completely—could be harmful or lethal because it would prevent the timely diagnosis of acute conditions needing immediate treatment,^20^ potentially leading to untreatable or severe conditions.^21^ Severe complications, unnecessary suffering for the individual, and a heavier burden on the healthcare system might be consequences of this strategy.^22^ The information given to rCDI patients about the risks of acquiring CDI needs to be balanced to avoid fostering unproductive and harmful preventive behaviours. This highlights the importance of giving personalized written information to patients and providing them with opportunities to calibrate their strategies with healthcare professionals based on written information.

The respondents gave rich descriptions of antibiotic treatment as a trigger for the onset of rCDI. This finding aligns well with the results of earlier research.^9^ The respondents claimed that they were careful and hesitant about using antibiotics after developing rCDI—a behaviour that could minimize future risks of rCDI, since the relationship between antibiotic exposure and the onset of disease is unquestionable.^23^ However, since antibiotics are an important tool in all modern medicine, it is essential that former rCDI patients are able to use them when necessary. The respondents also reported informing doctors about their rCDI histories and seeking expert opinions when needing antibiotic treatment. Since different antibiotics pose different risks of rCDI,^5^ such strategies could help patients minimize rCDI episodes in the future and, therefore, lighten the burden on the healthcare system. An effective information delivery process regarding how to use antibiotics in the future is crucial for rCDI patients and should be adopted by healthcare personnel.

Only one respondent saw a connection between in-hospital care and colonization by *C. difficile*,^24^ hesitating to use hospital toilets as a preventive behaviour to minimize the risk of rCDI. The respondents’ lack of awareness of the link between hospital care and the risk of colonization by *C. difficile* did not align with a Canadian survey^8^ showing that most of the study participants hesitated to seek hospital care because of the risk of acquiring CDI. This particular knowledge may not be of great importance for rCDI patients, since they are unlikely to be able to minimize the risk of colonization during hospital care by adopting preventive behaviours. This risk is best minimized by effective cleaning measures in hospitals.^25,26^ Therefore, this aspect of rCDI pathophysiology should not be stressed in the information provided to patients.

### Strengths and limitations

The study considered the perceptions of 10 rCDI patients from southwestern Sweden concerning the causes of their disease and the information given by healthcare staff. It provides important insights that can be used to underpin future studies, foster a better understanding of the interactions between patients and healthcare staff and raise awareness of the importance of a correct and standardized information delivery process in our setting. However, in settings with different healthcare systems and cultures, the generalizability of the results would be limited, although the study could be helpful in facilitating the understanding of information delivery processes in different environments.

We applied several procedures to ensure the trustworthiness of the results. The interview questions were open-ended, enabling the respondents to speak freely, and quotations were used to enhance the results and ensure a high level of credibility. By selecting the participants within an inclusion timeframe, we ensured that the respondents would not have an ongoing dependence on hospital staff that might bias their answers. We conducted two test interviews to ensure that the interview guide would promote open and honest communication during the interviews. We based the number of interviews on Brinkmann and Kvale,^27^ who argued that adding more and more participants generates less and less new knowledge. It is common practice in qualitative interview studies to include approximately 15 participants, with a variation of ±10. The qualitative content analysis was performed according to Elo and Kyngäs.^17^ After the first author conducted and transcribed the interviews and performed the first and second steps of the content analysis (selecting units of meaning and organizing the data), the first and last authors read the interview transcripts again to make sure that the analysis had captured the essence of the respondents’ perceptions. The last author also took an active part in the process of forming categories and subcategories so that this process was calibrated by two individual researchers.

### Conclusion

The findings of this study highlight the importance of information delivery processes, particularly objectivity-related and relationship-related strategies, in fostering productive preventive behaviours in patients suffering from rCDI. This knowledge could be an important asset to help patients avoid relapses and new rCDI episodes and therefore enhance their quality of life. Healthcare professionals should restore the trust of these patients by adopting effective information delivery processes, particularly by conveying correct information about one of the most important tools in modern medicine—antibiotics.
